# Obstetrics; the Science and Art

**Published:** 1857-03

**Authors:** 


ART. VII.—*Obstetrics: the Science and the Art.* By CHARLES D. MEIGS, M.D., Professor of Midwifery and the Diseases of Women and Children in Jefferson Medical College, Philadelphia, &c. &c. Third edition, 8vo. Philadelphia: Blanchard & Lea. 1856.

A WORK that has been so long before the profession, and so extensively read as that of Dr. Meigs, and more especially as its popularity is the reward of evident excellence, requires no notice at our hands beyond the simple announcement of a new edition. We may state, however, as a bit of personal history, that the author does not abate one jot or tittle from his peculiar views in regard to the "endangium," the nature of puerperal peritonitis, and some other subjects with which his name is so frequently associated, notwithstanding the great opposition which they have encountered both in this country and in Great Britain.

